# Semi-Synthetic Approach Leading to 8-Prenylnaringenin and 6-Prenylnaringenin: Optimization of the Microwave-Assisted Demethylation of Xanthohumol Using Design of Experiments

**DOI:** 10.3390/molecules25174007

**Published:** 2020-09-02

**Authors:** Corinna Urmann, Herbert Riepl

**Affiliations:** 1 Organic-Analytical Chemistry, Weihenstephan-Triesdorf University of Applied Sciences, 94315 Straubing, Germany; 2 Campus Straubing for Biotechnology and Sustainability, Technical University Munich, 94315 Straubing, Germany

**Keywords:** xanthohumol, 8-prenylnaringenin, 6-prenylnaringenin, demethylation, design of experiment, microwave synthesis

## Abstract

The isomers 8-prenylnaringenin and 6-prenylnaringenin, both secondary metabolites occurring in hops, show interesting biological effects, like estrogen-like, cytotoxic, or neuro regenerative activities. Accordingly, abundant sources for this special flavonoids are needed. Extraction is not recommended due to the very low amounts present in plants and different synthesis approaches are characterized by modest yields, multiple steps, the use of expensive chemicals, or an elaborate synthesis. An easy synthesis strategy is the demethylation of xanthohumol, which is available due to hop extraction industry, using lithium chloride and dimethylformamide, but byproducts and low yield did not make this feasible until now. In this study, the demethylation of xanthohumol to 8-prenylnaringenin and 6-prenylnaringenin is described the first time and this reaction was optimized using Design of Experiment and microwave irradiation. With the optimized conditions—temperature 198 °C, 55 eq. lithium chloride, and a reaction time of 9 min, a final yield of 76% of both prenylated flavonoids is reached.

## 1. Introduction

The isomers 8-prenylnaringenin (8PN) and 6-prenylnaringenin (6PN) are two representatives of prenylated flavonoids -secondary metabolites occurring in hops. 8PN, the most potent phytoestrogen known so far [1], shows a cytotoxic potential even in multi-resistant cancer cells [2]. Based on the estrogen-like activity of 8PN, this flavonoid is controversially discussed for use in hormone-replacement therapy [3,4,5]. The anti-cancer activity of 6PN is comparable to the activity of 8PN, whereby the lack of estrogen-like activity can be an advantage [6]. Although 6PN is less bioavailable than 8PN, it shows similar effects on the viability of peripheral blood mononuclear cells [7]. Furthermore, both compounds slightly induce differentiation in neural precursor cells and therefore can promote the development of neurons from stem cells [8]. These facts make it worthwhile to search for sources for these special flavonoids. Besides hops, both flavanones 6PN and 8PN were also isolated from *Psoralea corylifolia* [9] or different *Wyethia* spp. [10]. 8PN was furthermore identified in *Glycyrrhiza inflate*, *Sophora flavescens*, and other medicinal plants of Leguminosae and Moraceae [11]. In order to meet the needs, not the extraction but the synthetic production is recommended.

The synthesis of both flavonoids is characterized by modest yields, several steps using expensive chemicals or an elaborate synthesis. Nonselective prenylation of naringenin results in both flavanones in very low yields [12,13]. A targeted synthesis, also starting with naringenin and using metal catalysts, delivers 8PN [14] and 6PN [15] after six complex steps.

**Figure 1:**
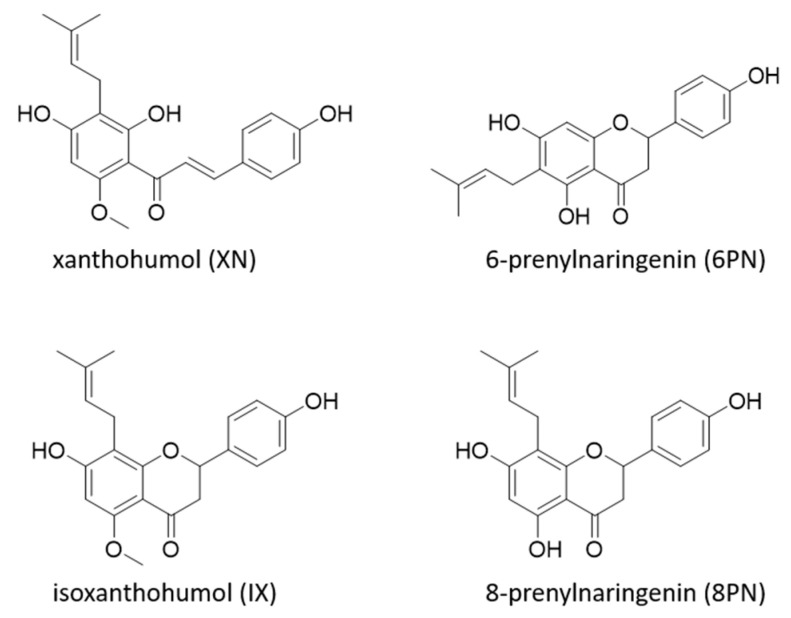
Structures of prenylated flavanones isoxanthohumol (IX), 8-prenylnaringenin (8PN), 6-prenylnaringenin (6PN), and the prenylated chalcone xanthohumol (XN).

Further approaches take advantage of the structural similarity of isoxanthohumol (IX) and 8PN (Figure 1). IX can be synthesized easily by alkaline isomerization of xanthohumol (XN), the most concentrated prenylflavonoid in hops, which is readily available due to the hop processing industry.

IX can be converted to 8PN using *Eubacterium limosum* [16,17] and *Eupenicillium javanicum* [18]. In addition to the biotechnological approach, scandium trifluoromethylsulfonate [19] or magnesiumiodide-etherat [20] were used for demethylation as synthetic strategies.

As a main problem of demethylation reactions, the formation of byproducts is identified [19]. This is due to a ring closure of the prenyl group or to additional products concerning the double bond [19]. This effect is particularly evident when using cheap salts like lithium chloride (LiCl) [19], although demethylation is quite easily performed with simpler compounds like aromatic methyl ethers [21]. The chloride ions are particular good nucleophiles in the underlying SN2-reaction due to the lack of hydrogen bonds [22]. A salt in combination with organic solvents is especially suitable for the use in a synthesis microwave due to the direct excitation of the ions [23]. The use of a higher pressure in a closed vessel system and therefore higher temperatures can accelerate reactions [24]. However, there are a lot of factors influencing the reaction. Accordingly, an excellent approach for the optimization of a microwave reaction is the “Design of Experiments” (DOE) approach, also known as multivariate analysis. The method changing “one factor at time” (OVAT) also called “classical method” considers experimental reaction factors one-dimensionally. This means that interactions between factors can be overlooked easily. Using DOE, several factors are systematically varied together at the same time and a mathematical model for the whole test space is implemented via regression functions. The results on the center points will be used several times and the identification of factor interactions is possible.

XN, the most abundant prenylflavonoid in hops, is available in relatively large amounts due to the hop extraction industry since it is retained in the plant residue during supercritical carbondioxide extraction. Consequently, XN is a good starting material for semi-synthetic approaches to obtain further prenylflavonoids. In this study, the microwave-assisted demethylation of XN leading to 8PN and 6PN was examined using DOE. The demands on the method investigated were thus the simultaneous production of 8PN and 6PN, while maintaining the prenyl group intact, a simple handling and an inexpensive execution due to inexpensive chemicals. To the best of our knowledge, this is the first report of a demethylation of XN leading to both flavanones 8PN and 6PN in similar yields.

## 2. Results and Discussion

**Figure 2:**
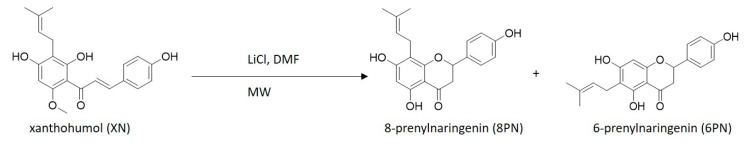
Demethylation of xanthohumol (XN) to 8-prenylnaringenin and 6-prenylnaringenin (6PN) using lithium chloride (LiCl), dimethylformamide (DMF), and microwave irradiation (MW).

Preliminary experiments showed that xanthohumol (XN) can be demethylated to 8-prenylnaringenin (8PN) and 6-prenylnaringenin (6PN) using lithium chloride, dimethylformamide, and microwave irradiation (Figure 2).

**Figure 3:**
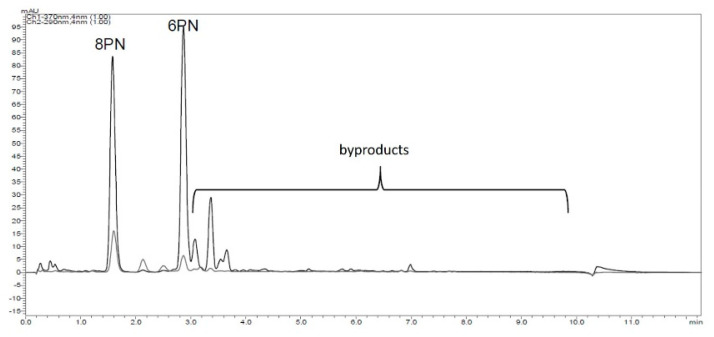
HPLC-chromatogram (λ = 290 nm flavanones; λ = 370 nm chalcones), with byproducts resulting from the reaction of XN with LiCl in DMF using microwave irradiation.

Using isoxanthohumol (IX) instead of XN, the selectivity of the reaction studied could not be improved. In addition, desmethylxanthohumol was observed in traces using LC/HRMS but disappeared during work up. This leads to the assumption that the flavanone ring of IX could be opened during the reaction and that the intermediate stage could be desmethylxanthohumol, leading to both flavanones via ring closing reaction. In contrast to Wilhelm et al., both flavanones were formed and not primarily 6PN [19]. Furthermore, the generation of byproducts, known from literature, was confirmed (Figure 3).

Accordingly, the best yield and purity can be reached, if the starting material XN is fully converted and low amounts of byproducts are formed, but the parameters to reach this are largely unknown. Since one advantage of DOE is the simultaneous observation and analysis of multiple targets, this approach was chosen to optimize the demethylation of XN. The temperature, the amount of LiCl, and the reaction time were investigated as factors with factor steps shown in Table 1.

A randomized central composite two step response surface plan with three factors in two blocks was created (Supplementary Material Table S1). Each block contained three central points and six rotatable star points. As targets, the yield of 8PN, 6PN, and the amount of XN at the end of the reaction were chosen and determined using HPLC-analysis and the absorption response. To quantify the amount of byproducts, the peak areas were analyzed (Figure 3).

**Figure 4:**
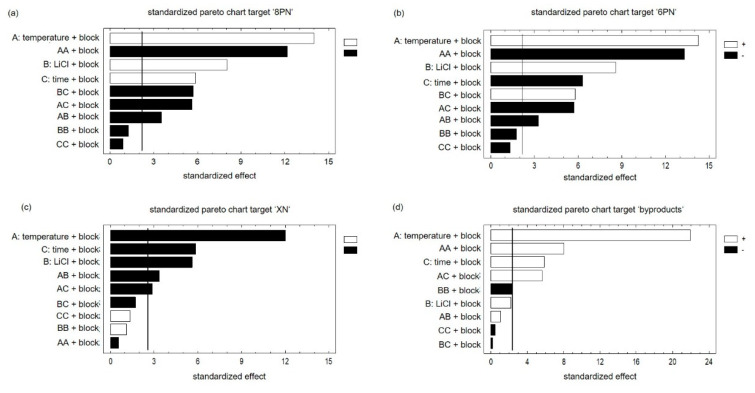
Pareto charts of all targets ‘8PN’ (**a**), ‘6PN’(**b**), ‘XN’(**c**) and byproducts (**d**) with a significance level of 5% concerning the factors ‘temperature’‚ ‘lithium chloride’, and ‘time’ as well as the ‘block factor’, which has to be considered due to the duration of the experiment.

Main effects and interactions did not correlate. Accordingly, the results could be interpreted without restriction. Using the probability net of residues, the normal distribution of the results was confirmed (Supplementary Material Figure S1). The comparison between observed and predicted results showed that the model is suitable (Supplementary Material Figure S2). In the next step, the effects of factors and their interaction were considered using the Pareto chart. The bars of the plot are in proportion to the calculated values of t-statistics of each effect. The vertical line indicates the error probability of 5% and divides the effects in significant (right) and not significant (left). Since the generation of both flavanones was influenced by the factors in the same way, the results of the target ‘8PN’ are exemplary discussed (Figure 4a,b).

The largest positive influence on the yield of 8PN is exerted by the factor ‘temperature’. Longer reaction times cause a transformation to byproducts and the reduction of product yield. The factor ‘LiCl’ as well as the factor ‘time’ showed a positive influence on the targets ‘8PN’ and ‘6PN’. All interactions of factors were significant, which only emphasizes the advantage of DOE and the need to consider the interactions of the factors. Accordingly, the optimum can only be determined considering all three factors. Since the star points were added at a later time point, a block factor has to be used. XN, the starting material, is consumed and is not formed again. Accordingly, all significant factors exhibit a negative influence on the target ‘XN’ (Figure 4c). The influences of all three factors were significant and the factor ‘temperature’ showed the greatest impact. Furthermore, the effect of the interactions of the factor ‘temperature’ with the other factors were significant. The opposite picture is observed at the target ‘byproducts’ (Figure 4d). A high temperature was conducive to the formation of byproducts. The factor ‘time’ also showed a significant influence on the formation of byproducts. It is worth mentioning that the factor ‘LiCl’ had no significant effect on the formation of secondary products.

The coefficient of determination R^2^ indicates the correlation between the calculated and experimental values of the chosen model and should be as close as possible to one (Table 2). In order to achieve comparability between different models, the R^2^ corrected on the degrees of freedom is usually used. Due to the values of R^2^_corrected_ greater than 90%, the model seems to be excellent for the prediction of reaction results.

**Figure 5:**
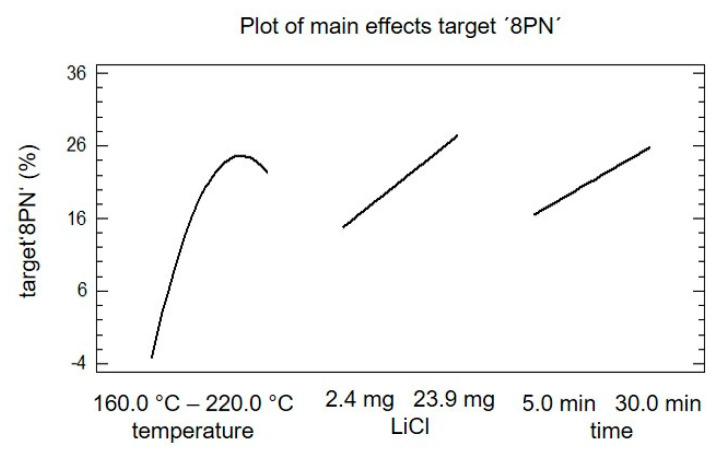
Plot of the main effects of target ‘8PN‘ concerning the factors temperature, lithium chloride, and time.

In Figure 5, the main effect plot of the target ‘8PN’ is shown. The slope of the different effects is proportional to the magnitude of the main effect and the sign indicates a positive or negative individual effect. The magnitude of the effect is defined as the difference of the mean values of the factor on the higher level and the lower level. All three factors show a significant effect on the target ‘8PN’. The individual analysis of the main effects indicates that an increase in the factor ‘LiCl’ and the factor ‘time’ would trigger an increase in yield of target ‘8PN’. The main effect of the factor ‘temperature’ showed a maximum; accordingly, the optimal temperature should be in the chosen experimental space.

**Figure 6:**
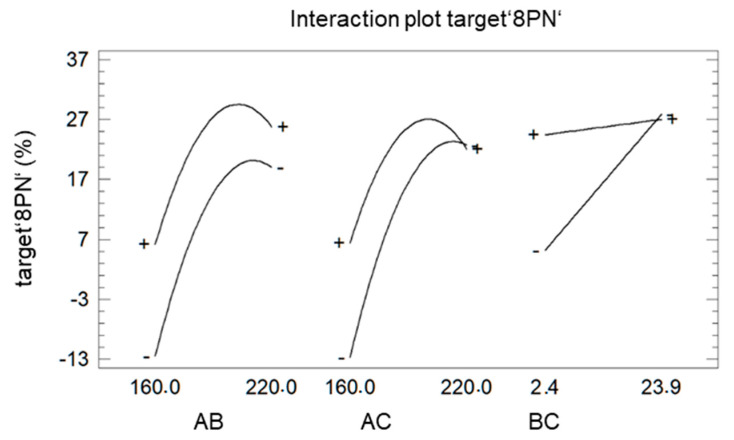
Interaction plot of target ‘8PN’ A: factor ‘temperature’ B: factor ‘LiCl’ C: factor ‘time’.

Since the representation as a Pareto chart (Figure 4) also revealed the significant interaction between the different factors, these interactions are further analyzed using the plot of interaction (Figure 6).

The deviation from parallelism indicates the strength of the effects. The factor ‘LiCl’ (B) showed a bigger impact on the target ‘8PN’ at a shorter (−) reaction time (C) than at longer reaction time (+). The influence of the factor ‘LiCl’ (B) at high and low temperature (A) is almost equal, indicating an advantage for increasing the factor ‘LiCl’ at both temperature settings. In case of a shorter reaction time (C), the maximum temperature of the factor ‘temperature’ shifts to larger values. This can be explained easily by the temperature dependence of the reaction rate.

**Figure 7:**
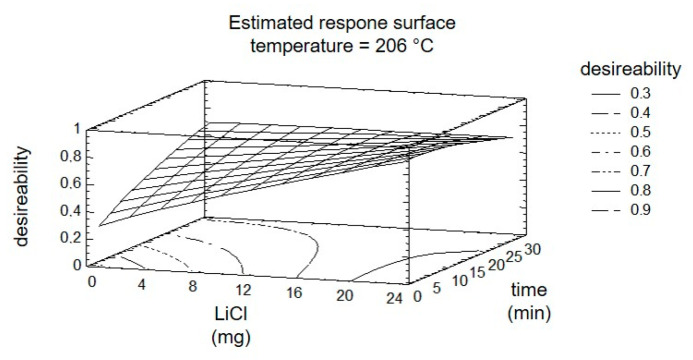
Estimated response surface fixing the temperature at 206 °C.

The subsequent optimization of the reaction by DOE considers all four targets and the three factors simultaneously. Using the response surface, shown in Figure 7, the results are summarized graphically. The entire experiment plan was aimed at maximizing the targets ‘8PN’ and ‘6PN’ and minimizing the targets ‘XN’ and ‘byproducts’. The yield maximization of the products was thereby weighted with a factor of two. The desirability, shown in the graphical evaluation (Figure 7), is defined as a value of the combination of factor settings that most effectively lead to the desired result and can take values between zero and one (optimal).

The factor temperature was set to the calculated optimum. The steepest increase of the response surface in the direction of greatest desirability is at short radiation time and an increasing factor ‘LiCl’. A control experiment with the settings at the calculated optimum showed a conformity of calculated and measured yields of 79% and 77%. The relatively poor match is probably due to the widely spaced levels of the factor time initially used.

Thus, an optimization in a smaller model range of reaction time was necessary in the next optimization step. A new experimental design was set up around the optimum values (Supplementary Material Table S2). The full factorial central composite randomized response surface plan with five centrum points is shown in the Supplementary Material and the factor steps are shown in Table 3.

The data still came from a normal distributed population and the fit of the model was still given. The only difference in the Pareto chart was that, in contrast to the first experimental design, the interactions with the participation of the factor ‘LiCl’ were no longer significant. The coefficient of determination R^2^ and R^2^_corrected_ continued to show a good agreement between the calculated and the experimentally determined values. The plot of the main effects showed the same picture as already discussed above. Both the increase of factor ‘LiCl’ as well as the factor ‘time’ led to an increase in the yield of 8PN. At shorter reaction time, the factor temperature showed a stronger influence on the yield compared to longer reaction time, which remains the only significant interaction (plots are shown in the Supplementary Material Figures S3 and S4).

**Figure 8:**
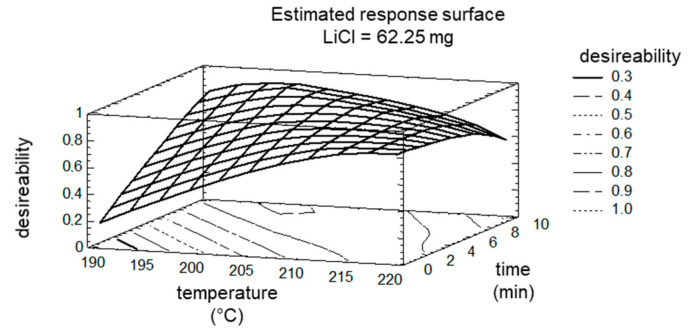
Estimated Response Surface; the factor LiCl is fixed at 65.25 mg.

The optimum was calculated to still fit the maximization of target ‘8PN’, ‘6PN’, and ‘XN’ (maximization of conversion) and the minimization of target ‘byproducts’. The comparison of the calculated optimum with the measured values of the control experiment showed a very good agreement of 97%. The good agreement can be attributed to the narrow limits of the factors. Even if the factor ‘LiCl’ was no longer determined to be significant, the DOE plan was expanded to higher amount of LiCl (65.25 mg), since this factor was the only one without a maximum in the experimental space tested. Actually, this also did not result in a maximum of factor ‘LiCl’, but the impact on targets ‘8PN’ and ‘6PN’ were only marginal. The maxima/optima of factor ‘time’ and factor ‘temperature’ are shown in the estimated response surface (Figure 8).

The comparison of the calculated yield versus measured values at the optimal settings was tested in triplicate. The reproducibility of the results is given with a standard deviation of 0.85% and 0.88% or in other words with an agreement of 99% (Table 4).

The demethylation of XN to 8PN and 6PN could be optimized to an overall yield of 76% (HPLC) using DOE. The byproducts, which were created for instance by cyclization reaction of prenyl group with neighboring OH-group are well known [19,25,26,27], but could be mostly avoided using LiCl in neutral conditions [21] and optimizing the reaction. Demethylation using LiI in pyridine was not successful [19] and almost the same low yields can be reported in DMF using the optimized conditions. Using Lewis acids like AlBr_3_ for demethylation of IX, a yield of 30% of 8PN was reached, but only traces of 6PN [19]. If only 8PN is required, a yield of 92% could only be achieved by demethylation using scandium triflate in THF [19], which is approximately 300 times more expensive than LiCl. A demethylation of XN leading to good yields of 6PN is not reported so far and the full synthesis consists of seven steps including cross metathesis [15].

The disadvantages of LiCl like low activity, unsatisfactory yields, and longer reaction times, which limited its practical application could be avoided using microwave irradiation [28]. Furthermore, LiCl is a significant factor influencing the selectivity and reaction rate of the demethylation, which is also known from OVAT experiments [21]. However, in the end, the comparison of the optimized closed-vessel microwave method with conventional heating in a closed and open vessel was of interest, since not every lab has a synthesis microwave. The closed vessel reaction has been first carried out with the optimal settings of DOE, which results in a yield of 4% compared to 38% using microwave irradiation. To take the heating time of the microwave irradiation into account, the reaction time then was extended to 14.34 min resulting in a yield of almost 19%. Since the yields were not comparable to those of the microwave reaction, time span was extended to 300 min, and a full conversion of XN could be achieved and led to yields of approximately 35%, which are comparable to the yield of the microwave reaction. The optimized reaction time can obviously not be transferred to a conventional execution. In this context, the term microwave-specific enhancement is often used. However, a simple acceleration of reaction time is not sufficient to declare a microwave-specific influence [24,29] and accordingly this question should be addressed in future studies.

Usually, three to five equivalents of LiCl are used [21,28] and raising the equivalents of LiCl also accelerated the reaction under conventional heating in this study.

Using an open vessel for the reaction, the temperature used is limited to 153 °C due to the boiling point of DMF. Since reaction temperature is lower, the reaction rate should be lower [23] and usual reaction times for demethylation of alkyl aryl ethers are around 20 to 72 h [28]. Demethylation of XN to 8PN and 6PN is possible in an open vessel system with a condenser, but a reaction time of 300 min was not sufficient for good yields. This is in good accordance to literature, since the execution of the demethylation reaction of aryl ethers using conventional refluxing gave lower yields after a 40 fold reaction time than using microwave irradiation [21].

In summary, combining the advantages of microwave synthesis and a statistical design of experiment approach, an efficient demethylation of xanthohumol using LiCl in DMF leading to 8-prenylnaringenin and 6-prenylnaringenin was developed. The use of compounds with estrogen-like activity in medicine are discussed controversial, due to the increasing risk of carcinogenic events [3,4,5]. Accordingly, the low estrogen-like activity of 6PN compared to 8PN [1,11,30,31,32] can be an advantage, and easy and cheap methods to get 6PN are beneficial.

By optimization of the reaction conditions both flavanones could be obtained with a total yield of 76% and a formation of byproducts could be suppressed. The developed method offers further advantages, including a rapid reaction rate, use of inexpensive reagents, and a simple execution.

## 3. Materials and Methods

### 3.1. Microwave System

Microwave irradiation was carried out with CEM Discover S-class single-mode synthesis system interfaced with a laptop PC running CEM synergy software monitoring the reaction. The temperature was checked by an external infrared sensor in the bottom of the cavity. Once the target temperature was reached, the microwave system automatically started to count down the hold time. For reactions, CEM vials of 10 mL with snap-on caps were used. The pressure was monitored by a sensor outside the snap-on caps. The upper pressure limit was set to 18 bar. Temperature/pressure recording were attached to CEM synergy reaction files.

### 3.2. Microwave Reaction

The respective amount of lithium chloride was weighed in a 10 mL microwave reaction vessel and then mixed with 1 mL of a stock solution of xanthohumol (c = 10 mg/mL) in anhydrous dimethylformamide. The reaction mixture was irradiated for the indicated times, wherein the time only started upon reaching the reaction temperature. The reaction mixture was mixed with 2 mL water and 200 µL hydrochloric acid (c = 2 mol/L) and extracted using 2 mL ethyl acetate. In addition, 1 mL of the organic layer was taken and the solvent was removed in vacuum. Afterwards, it was soluted in 25 mL acetonitrile, filtered through a PTFE filter, and analyzed using HPLC/PDA.

### 3.3. Synthesis Reference Compounds

Xanthohumol was purified using preparative HPLC from a hop extract according to [8]. The reference compounds 8-prenylnaringenin and 6-prenylnaringenin were synthesized according to [14] and [15]. The analytical data correspond to that of literature.

### 3.4. HPLC-Method

For HPLC-analytics, a Shimadzu system (2xLC-20AD, SSIL-20AC HT, CTO-20A, SPD-M20) using a column (Phenomenex, Kinetex C_18_ 4.6 × 50 mm, 2.6 u, 100 Å) and the following method with A (water, 1% formic acid) and B (acetonitrile); flow 3 mL/min: 00.00–0.30 min 35% B, 0.31–1.00 min 35–41% B, 1.01–8.08 min 43–91% B; 8.09–8.30 min 91–95% B; 8.31–8.81 min 95% B was used. The wavelength of the PDA-detector was set to 290 nm (flavanones) and 370 nm (chalcones).

### 3.5. Statistics

HPLC: Calibrations curves of 8-prenylnaringenin, 6-prenylnaringenin, and xanthohumol were carried out in range of 0.06 mg/mL–0.6 mg/mL. The calibration curve (R = 0.999) was analyzed using SQS 2000 © Dr. Joachim Kleiner. The limit of quantification of 8-prenylnaringenin was 0.014 mg/mL, 6-prenylnaringenin 0.018 mg/mL, and of xanthohumol 0.06 mg/mL. The amount of byproducts was evaluated using peak areas especially by addition of peak areas straight behind 6-prenylnaringenin until R_T_ = 10 min without the area of xanthohumol.

DOE: Statgraphik^®^ (StatPoint) was used for experimental design and evaluation. The actual weighed amount of lithium chloride was considered for the test evaluation. Not practicable rotable star points were converted into face-centered star points. Values which were under the limit of quantification and determination were removed from the DOE model.

## Figures and Tables

**Table 1:** Factor steps of the experiment plan of the first DOE approach.

Step.	A Temperature °C	B LiCl mg	C Time min
low	160	2.4	5
high	220	23.9	30

**Table 2:** Coefficient of determination R^2^ and corrected coefficient of determination R^2^_corrected_ concerning the targets.

Target	R^2^ (%)	R^2^_corrected_
8PN	0.951	0.929
6PN	0.947	0.923
XN	0.950	0.924
By products	0.986	0.977

**Table 3:** Factor steps of the second experimental plan.

Step	A Temperature °C	B LiCl mg	C Time min
low	190.0	37.1	1.0
high	220.0	55.87	9.0

**Table 4:** Comparison of the calculated with measured values at optimal settings.

	Temperature °C	LiCl mg	Time min	Yield 8PN %	Yield 6PN %
Calculated optima	198	65.35	9.34	38.0	38.2
Experimental results	198	65.75	9.34	37.6	37.6
Average ± standard deviation				37.7 ± 0.85 %	38.4 ± 0.88 %

## Supplementary Materials

The following are available online: Figure S1. Plot observed versus predicted values for the target ‘8PN’. Figure S2. Interaction plot of target ‘8PN’. Figure S3. Standardized Pareto charts for targets ‘8PN’, ‘6PN’ and ‘XN’. Figure S4. Interaction plot of target ‘8PN’ Table S1. Experimental plan 1. Table S2. Experimental plan 2.
