# Supplementary material for: Semi-Synthetic Approach Leading to 8-Prenylnaringenin and 6-Prenylnaringenin: Optimization of the Microwave-Assisted Demethylation of Xanthohumol Using Design of Experiments
Source: Molecules. 2020 Sep 2;25(17):4007. doi: 10.3390/molecules25174007 (PMC7504789; doi:10.3390/molecules25174007)
Supplement: Supplementary file 1 [file molecules-25-04007-s001.pdf]

Supplementary Material

Semi-synthetic approach leading to 8-prenylnaringenin and 6-prenylnaringenin: Optimization of the microwave-assisted demethylation of xanthohumol using Design of Experiments

Corinna Urmann [1,2*], and Herbert Riepl [1,2*]

[1] Organic-analytical Chemistry, Weihenstephan-Triesdorf University of Applied Sciences, Straubing, Germany ; corinna.urmann@hswt.de; herbert.riepl@hswt.de

[2] Campus Straubing for Biotechnology and Sustainability, Technical University Munich, Straubing, Germany; corinna.urmann@tum.de

* Correspondence: corinna.urmann@tum.de; Tel.: +49-9421-187229; herbert.riepl@hswt.de Tel: +49-9421-187302

Figure S 1. plot observed versus predicted values for the target ‘8PN’. 2
Figure S 2. interaction plot of target ‘8PN’. 2
Figure S 3. standardized pareto charts for targets '8PN', '6PN' and 'XN’. 3
Figure S 4. interaction plot of target ‘8PN’ 3

Table S 1. Experimental plan 1 1
Table S 2. Experimental plan 2 2

Table S 1. Experimental plan 1 and maximum pressure.

Block	Temperatur (°C)	LiCl (mg)	Time (min)	Maximum Pressure (bar)
1	190	13.4	17.5	3
1	160	23.77	30	0
1	160	2.54	5	0
1	220	2.5	5	6
1	190	13	17.5	3
1	160	2.6	30	1
1	190	13.46	17.5	3
1	220	23.8	30	7
1	220	23.9	5	7
1	160	24	5	0
1	220	2.6	30	7
2	190	13.2	5	3
2	190	2.32	17.5	3
2	135	13.06	17.5	0
2	190	13.19	40	3
2	190	13.23	17.5	3
2	244	13.49	17.5	9
2	190	32.32	17.5	4
2	190	13.14	17.5	3
2	190	13.29	17.5	3

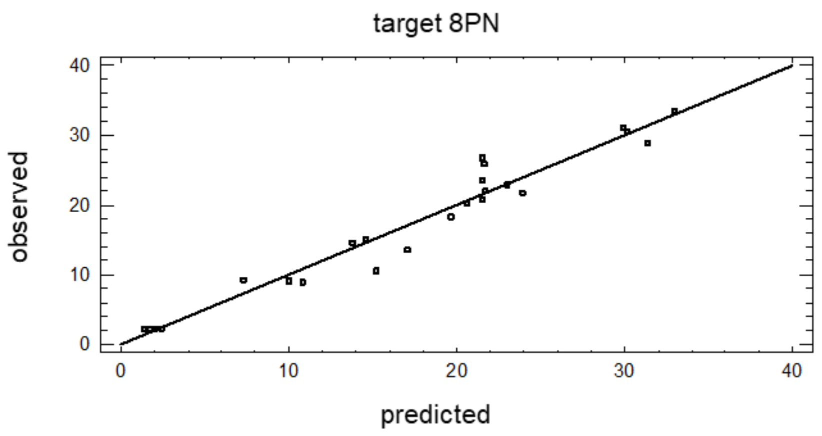

Figure S 1. Plot observed versus predicted values for the target '8PN'.

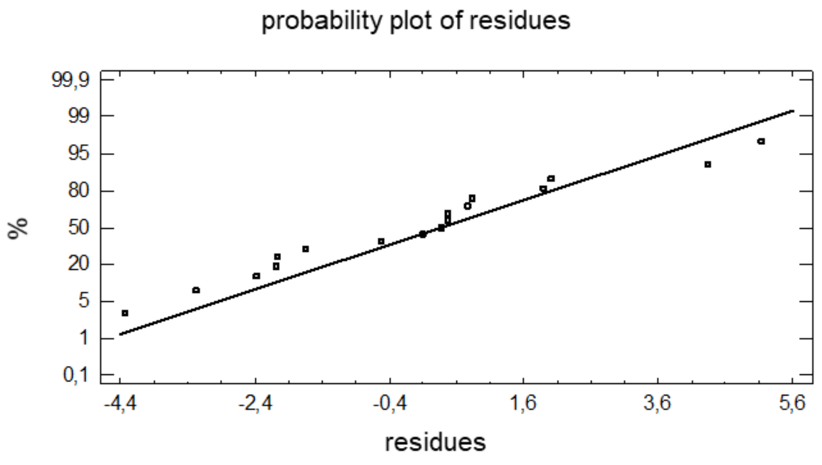

Figure S 2. Interaction plot of target '8PN'.

Table S 2. Experimental plan 2 and maximum pressure.

Block	Temperatur (°C)	LiCl (mg)	Time (min)	Maximum Pressure (bar)
1	205	36.81	1	4
1	190	47.89	1	3
1	205	36.79	5	4
1	205	37.18	5	4
1	205	37	5	6
1	220	47.55	9	6
1	180	37.13	5	2
1	190	26.98	1	3
1	190	47.65	9	3
1	205	36.63	11.7	5
1	205	37.45	5	4
1	220	47.83	1	6
1	205	37.3	5	5
1	220	26.25	1	8

1	205	54.87	5	4
1	205	19.79	5	6
1	220	26.54	9	8
1	230	37.41	5	9
1	190	26.91	9	3
2	200	55.87	11.7	4
3	200	65.35	7.27	4

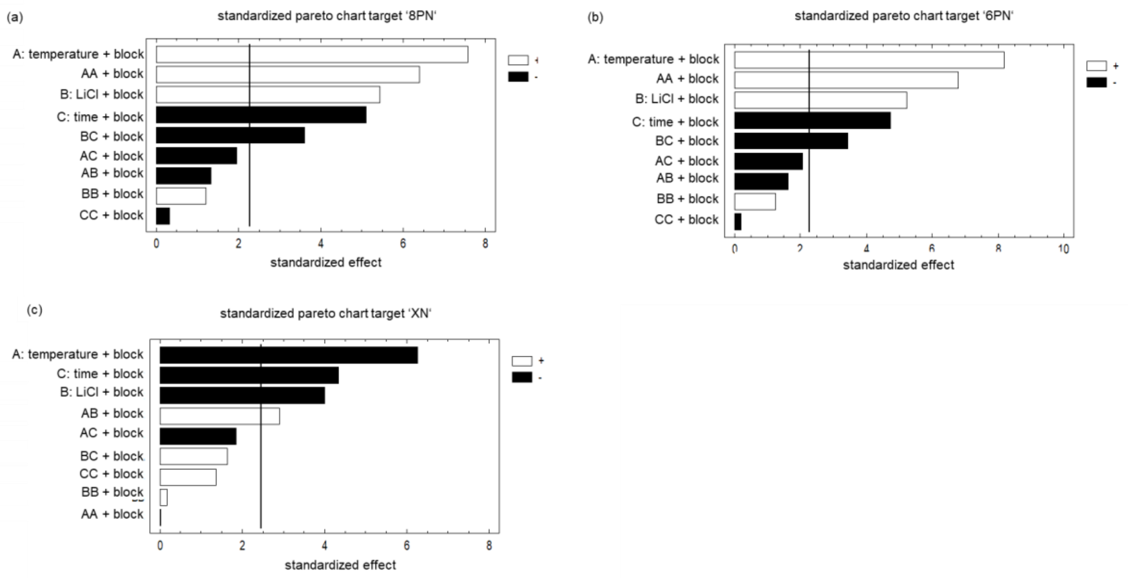

Figure S 3. Standardized pareto charts for targets '8PN', '6PN' and 'XN'.

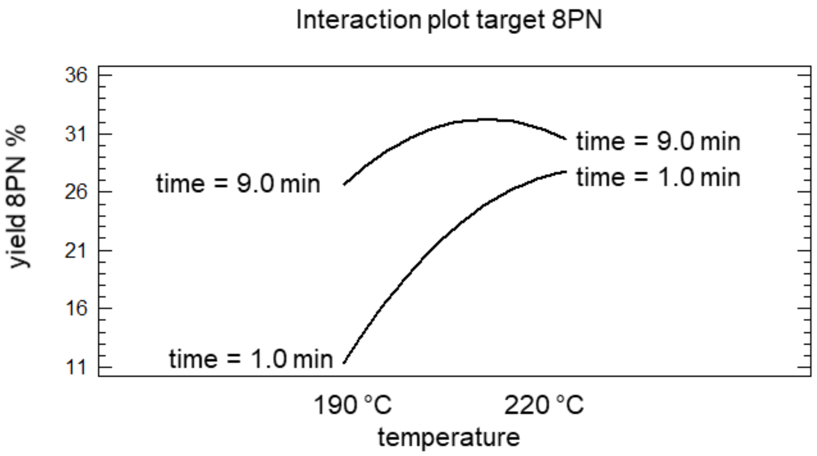

Figure S 4. Interaction plot of target '8PN'
